# The Current Status of Secondary Use of Claims, Electronic Medical Records, and Electronic Health Records in Epidemiology in Japan: Narrative Literature Review

**DOI:** 10.2196/39876

**Published:** 2023-02-14

**Authors:** Yang Zhao, Tadashi Tsubota

**Affiliations:** 1 Audit & Assurance Deloitte Analytics R&D Deloitte Touche Tohmatsu LLC Tokyo Japan

**Keywords:** claims, electronic medical records, EMRs, electronic health records, EHRs, epidemiology, narrative literature review

## Abstract

**Background:**

Real-world data, such as claims, electronic medical records (EMRs), and electronic health records (EHRs), are increasingly being used in clinical epidemiology. Understanding the current status of existing approaches can help in designing high-quality epidemiological studies.

**Objective:**

We conducted a comprehensive narrative literature review to clarify the secondary use of claims, EMRs, and EHRs in clinical epidemiology in Japan.

**Methods:**

We searched peer-reviewed publications in PubMed from January 1, 2006, to June 30, 2021 (the date of search), which met the following 3 inclusion criteria: involvement of claims, EMRs, EHRs, or medical receipt data; mention of Japan; and published from January 1, 2006, to June 30, 2021. Eligible articles that met any of the following 6 exclusion criteria were filtered: review articles; non–disease-related articles; articles in which the Japanese population is not the sample; articles without claims, EMRs, or EHRs; full text not available; and articles without statistical analysis. Investigations of the titles, abstracts, and full texts of eligible articles were conducted automatically or manually, from which 7 categories of key information were collected. The information included organization, study design, real-world data type, database, disease, outcome, and statistical method.

**Results:**

A total of 620 eligible articles were identified for this narrative literature review. The results of the 7 categories suggested that most of the studies were conducted by academic institutes (n=429); the cohort study was the primary design that longitudinally measured outcomes of proper patients (n=533); 594 studies used claims data; the use of databases was concentrated in well-known commercial and public databases; infections (n=105), cardiovascular diseases (n=100), neoplasms (n=78), and nutritional and metabolic diseases (n=75) were the most studied diseases; most studies have focused on measuring treatment patterns (n=218), physiological or clinical characteristics (n=184), and mortality (n=137); and multivariate models were commonly used (n=414). Most (375/414, 90.6%) of these multivariate modeling studies were performed for confounder adjustment. Logistic regression was the first choice for assessing many of the outcomes, with the exception of hospitalization or hospital stay and resource use or costs, for both of which linear regression was commonly used.

**Conclusions:**

This literature review provides a good understanding of the current status and trends in the use of claims, EMRs, and EHRs data in clinical epidemiology in Japan. The results demonstrated appropriate statistical methods regarding different outcomes, Japan-specific trends of disease areas, and the lack of use of artificial intelligence techniques in existing studies. In the future, a more precise comparison of relevant domestic research with worldwide research will be conducted to clarify the Japan-specific status and challenges.

## Introduction

### Background

Medical claims data, electronic medical records (EMRs), and electronic health records (EHRs) are familiar sources of real-world data (RWD). They are often used secondarily to complement limitations in clinical trials. For example, they can characterize patient subgroups that are excluded from clinical trials by following eligibility criteria such as comorbidities or age. Findings obtained through long-term, naturalistic observations of a large and diverse patient population can be easily generalized to other populations. Other advantages are that these data have high external validity, a single data source can be used for different study purposes, and prospective data collection is not required.

Claims data are electronic records of transactions between patients and health care providers. They include information on bills (claims) submitted by providers (hospitals, clinics, and pharmacies) to third-party payers (health insurance associations). There are already some large-scale commercial and nonprofit claims databases available in Japan [1-6] that aggregate information from multiple health care providers for secondary use**.** Recently, the EMR and EHR data have become widely available. The EMR data are the details of the encounters with patients recorded by physicians through EMR systems. They contain rich clinical information such as laboratory test results, diagnostic images, pathology findings, and patient symptoms. As different facilities may use different EMR systems, domestic EMR data are currently available from ≥1 medical institution. The EHR data are electronic records of all health-related information of individual patients created and managed by clinical professionals, which can be shared and used among various medical facilities. Current EHR databases in Japan include both patient claims data and medical records.

In recent years, claims data, EMRs, and EHRs have been increasingly used in clinical epidemiology studies. Such studies include cost-effectiveness analysis of drugs (including disease burden and assessment of medical technology), risk factor analysis, investigation of the actual status of drugs (including preclinical feasibility valuation, marketability study, and detection of prescription patterns), and evaluation of drug efficacy in actual clinical practice. Because these data are not designed for research purposes, the secondary use requires an understanding of their limitations and the ability to generate clinical questions, epidemiological skills to construct a study design, and statistical skills to analyze retrospective observational data. Previous approaches have addressed the limitations and challenges of using these data [7-12]. Understanding their application status based on these advanced guidelines is essential. However, investigations of existing epidemiological studies based on these data are lacking.

### Objective

We conducted a comprehensive narrative literature review to clarify the secondary use of claims, EMRs, and EHRs in clinical epidemiology in Japan. We focused on 7 categories of key information, including organization, study design, RWD type, database, disease, outcome, and statistical method. We expect that this review would help in the design of high-quality epidemiological studies.

## Methods

### Overview

This is a comprehensive narrative literature review that investigated the secondary use of claims data, EMRs, and EHRs in epidemiology in Japan. Referring to PRISMA (Preferred Reporting Items for Systematic Reviews and Meta-Analyses) guidelines [13] and procedures used in previous review studies [14-18], we conducted this review by searching for biomedical articles in PubMed.

### Information Source

We searched peer-reviewed publications that satisfied the eligibility criteria for this narrative literature review in PubMed from January 1, 2006, to June 30, 2021 (the date of search).

### Search Strategy

Keywords used to search PubMed consisted of “real world,” “database,” “claim,” “receipt,” “administrative,” “emr,” “ehr,” “japan,” “electronic medical record,” “electronic health record,” and Medical Subject Headings (MeSH) terms including, “Electronic Health Record,” “Administrative Claims, Healthcare,” “Insurance Claim Review/statistics and numerical data,” and “Japan/epidemiology.” We initially identified related articles by using various combinations of these keywords. The details of the search string are available in Multimedia Appendix 1.

### Eligibility Criteria

On the basis of the search strategy, we identified articles whose titles and abstracts satisfied the following three inclusion criteria: (1) involvement of claims, EMRs, EHRs, or medical receipt data; (2) mention of Japan; and (3) published from January 1, 2006, to June 30, 2021. Eligible articles were then filtered out by satisfying any of the following six exclusion criteria: (1) review articles; (2) non–disease-related articles; (3) articles in which the Japanese population is not the sample; (4) articles without claims, EMRs, or EHRs; (5) unavailability of full-text articles; and (6) articles without statistical analysis.

### Selection Process

The second author (TT) conducted the article search based on the search strategy. Both authors jointly reviewed all searched publications and performed 2 rounds of screening to identify target eligible articles. In the first round, we removed duplicates and articles that met any of the 6 exclusion criteria by screening the titles and abstracts. Review articles were automatically identified by a section classification model [19] trained on the PubMed 200k data set [20], which classified sentences in the abstracts into 5 sections (introduction, objective, method, result, and conclusion). On the basis of the hypothesis that review articles do not have sentences describing the results, we considered those without result sentences as review articles and removed them from the target articles. Artificially, we filtered out articles that met the exclusion criteria (2)-(5). In the second round of screening, the first author (YZ) reviewed the full text of the remaining articles and removed those that did not include statistical analysis. The 2 authors double checked the results to ensure accuracy and finalized the eligible articles.

### Data Collection

#### Overview

Investigations of the titles, abstracts, and full texts were conducted for eligible articles, from which 7 categories of key information were collected. The information included organization, study design, RWD type, database, disease, outcome, and statistical methods. Details regarding the classifications for each category are provided in Multimedia Appendix 2.

#### Automated Data Extraction

Four of these categories, including organization, study design, RWD type, and disease, were automatically extracted by keywords matching on the titles and abstracts. Two authors coded the data collection together.

On the basis of authors’ address information, organization was classified into 3 groups: “academic,” “nonacademic,” and “collaboration,” which denote that a study was conducted by academia, enterprises (including pharmaceutical companies, biotechnology companies, medical device companies, voluntary associations, and other health care–related companies), or collaboration of academia and nonacademic enterprises, respectively. Study design information was extracted by matching sentences in the abstracts to the categories listed in Multimedia Appendix 2, which consists of cohort studies, case-control studies, case-crossover studies, and cross-sectional studies. Similarly, RWD-type information was extracted by matching sentences in the abstract with 3 keywords, including claims, EMRs, and EHRs. Disease information was classified according to tree codes C01-C26 of MeSH terms [21]. For articles without the corresponding MeSH terms, disease information was collected from their titles using MetaMap [22] and pyMeSHSim [23].

#### Manual Data Extraction

Subsequently, the first author (YZ) conducted a full-text investigation to collect information on the database, outcome, and statistical method used in the target articles. The second author (TT) cross-checked the results of this data collection.

**Figure 1:**
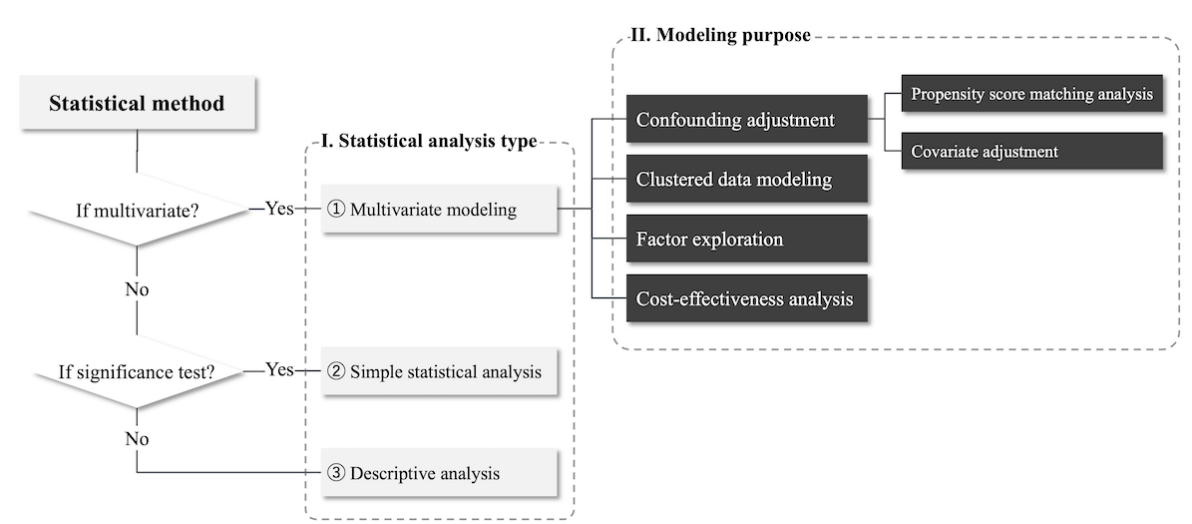
A hierarchical approach for collecting information on the statistical method.

Database information was collected directly from the full texts. For those articles that did not use a specific database, we categorized them uniformly according to their data source as “other database” or “municipal claims database,” where “other database” indicates data from 1 or more medical facilities and “municipal claims database” indicates claims data provided by regional administrative agencies. Because there is no familiar way of categorizing outcomes for RWD studies, we defined 8 classifications of outcomes by referring to the article by Abaho et al [24]. The explanations for these classifications are detailed in Multimedia Appendix 2. We defined a hierarchical approach to collect information on statistical methods in the text. As shown in Figure 1, the method used in these articles was first categorized as multivariate modeling, simple statistical analysis, or descriptive analysis. Then, multivariate modeling was subdivided according to the purposes of confounding adjustment, clustered data modeling, factor exploration, or cost-effectiveness analysis, where confounding adjustment was further classified according to whether propensity score (PS) analysis was conducted.

It should be noted that an article that focuses on multiple diseases, RWD types, study designs, databases, outcomes, or modeling purposes would be double counted for each classification to which it belongs.

### Analysis

We performed a descriptive statistical analysis of the collected data by describing their counts and percentages. In addition, we calculated the percentages of outcomes and databases for each disease. The percentages of statistical methods used to assess different outcomes were also analyzed. All codes used for data collection and descriptive analyses were performed using Python (version 3.8.8, 2021).

## Results

### Study Selection

**Figure 2:**
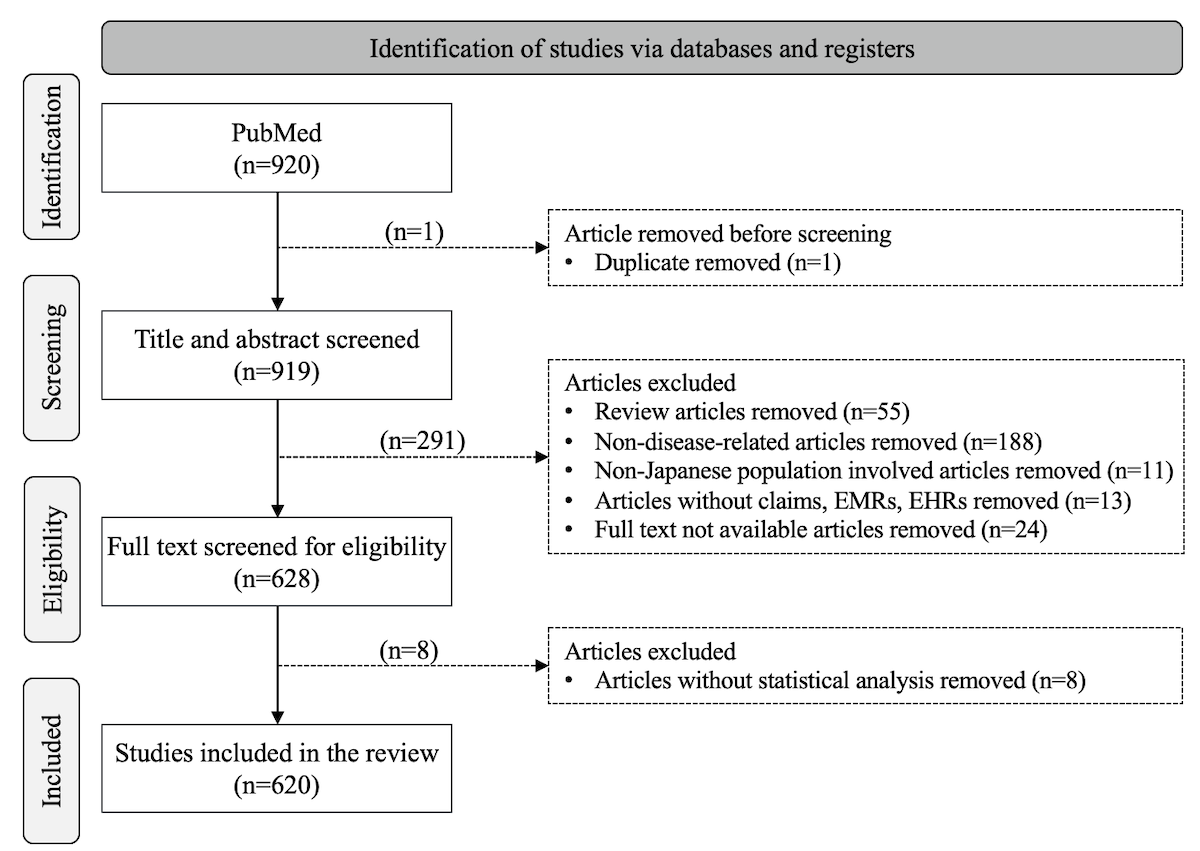
Search and screening process [13-18]. EHR: electronic health record; EMR: electronic medical record.

A total of 620 eligible articles were identified for this narrative literature review. Figure 2 [13-18] illustrates the selection process and the results of each screening step. We also illustrate the publication years of these articles in Multimedia Appendix 3. The distribution indicated that 68.7% (426/620) of the articles were published after 2018, suggesting that the secondary use of the 3 RWD types in epidemiological research in Japan was prevalent in approximately the last 5 years.

### Summary of Findings

#### Overview

We summarize the counts and percentages of information in the 7 categories and illustrate the top-ranked items for each category in Tables 1 and 2. All results are detailed in Multimedia Appendix 4. It should be noted that for an article with multiple diseases, data types, study designs, databases, outcomes, or modeling purposes, it was double counted in each classification to which it belongs. Therefore, the total percentage of these categories may not be 100%. The following subsections present the results for each category.

**Table 1:** Results of counts and percentages of the 7 categories (n=620).

Category		Count, n (%)
**Organization**		
	Academic	429 (69.2)
	Nonacademic	153 (24.7)
	Collaboration	35 (5.6)
**Study design**		
	Cohort study	533 (86)
	Case-control study	30 (4.8)
	Case-crossover study	23 (3.7)
	Cross-sectional study	6 (1)
**RWD^a^ type**		
	Claim	594 (95.8)
	EMR^b^	30 (4.8)
	EHR^c^	4 (0.6)
**Database**		
	JMDC^d^	181 (29.2)
	DPC^e^ database (MHLW^f^)	141 (22.7)
	MDV^g^	103 (16.6)
	NDB^h^	65 (10.5)
	Other databases	26 (4.2)
	JROAD-DPC^i^	17 (2.7)
	Municipal claims database	12 (1.9)
	QIP^j^	10 (1.6)
**Disease**		
	Infections	105 (16.9)
	Cardiovascular diseases	100 (16.1)
	Neoplasms	78 (12.6)
	Nutritional and metabolic diseases	75 (12.1)
	Digestive system diseases	68 (11)
	Pathological conditions, signs and symptoms	63 (10.2)
	Nervous system diseases	62 (10)
	Musculoskeletal diseases	42 (6.8)
	Mental disorders	38 (6.1)
	Wounds and injuries	33 (5.3)
	Male urogenital diseases	30 (4.8)
	Respiratory tract diseases	27 (4.4)
	Hemic and lymphatic diseases	16 (2.6)
	Eye diseases	14 (2.3)
	Skin and connective tissue diseases	10 (1.6)
**Outcome**		
	Treatment patterns	218 (35.2)
	Physiological or clinical	184 (29.7)
	Mortality	137 (22.1)
	Resource use or costs	118 (19)
	Hospitalization or hospital stay	107 (17.3)
	Adverse events	97 (15.6)
	Guideline adherence	32 (5.2)
	Quality indicators	5 (0.8)
**Statistical method**		
	Multivariate modeling	414 (66.8)
	Simple statistical analysis	121 (19.5)
	Descriptive analysis	85 (13.7)

^a^RWD: real-world data.

^b^EMR: electronic medical record.

^c^EHR: electronic health record.

^d^JMDC: Japan Medical Data Center Claims.

^e^DPC: diagnosis procedure combination.

^f^MHLW: Ministry of Health, Labour and Welfare.

^g^MDV: medical data vision.

^h^NDB: National Database of Health Insurance Claims and Specific Health Checkups of Japan.

^i^JROAD-DPC: Japanese Registry of All Cardiac and Vascular Disease-diagnosis procedure combination.

^j^QIP: Quality Indicator/Improvement Project.

**Table 2:** Results of modeling purposes as defined in Figure 1 and specific models used in the 414 multivariate modeling studies.

Category of multivariate modeling studies			Count (n=414), n (%)
**Modeling purpose**			
	**Confounding adjustment**		375 (90.6)
		Propensity score matching analysis	96 (23.2)
		Covariate adjustment	279 (67.4)
	Clustered data modeling		69 (16.7)
	Factor exploration		68 (16.4)
	Cost-effectiveness analysis		8 (1.9)
**Specifical method**			
	Logistic regression		249 (60.1)
	Cox proportional hazards regression		87 (21)
	Linear regression		57 (13.8)
	Poisson regression		23 (5.6)
	GLM^a^		18 (4.3)

^a^GLM: generalized linear model.

#### Organization

In Table 1, the results of organization show that most (429/620, 69.2%) target articles were conducted by academics, whereas nonacademic firms preferred to collaborate with academic institutions (153/620, 24.7%).

#### Study Design

The results of study design show 86% (533/620) of the articles that performed cohort studies, whereas only a few (30/620, 4.8%) studies were case-control studies, cross-sectional studies (23/620, 3.7%), and case-crossover studies (6/620, 1%).

#### RWD Type

Most (594/620, 95.8%) studies used claims data. Only a small number (30/620, 4.8%) of studies used EMRs and (4/620, 0.6%) EHRs. According to the articles that used EMRs or EHRs, we found that these studies commonly collected EMRs or EHRs from private databases (1 or some specific hospitals), which did not have large patient populations.

#### Database

Table 1 shows the top-ranked databases (n≥10) used in the target articles. The Japan Medical Data Center Claims (JMDC) database, a well-known, large-scale commercial insurance-based claims database operated by JMDC Inc [3,4], was the most used database. JMDC was used in 29.2% (181/620) of the total articles. The second most used database is composed of claims data from diagnosis procedure combination (DPC) hospitals provided by the Ministry of Health, Labour and Welfare (MHLW) [25,26], which we called the DPC database (MHLW). A total of 22.7% (141/620) of articles used the DPC database (MHLW). Medical data vision (MDV) [5], another commercial hospital claims-based database, was used for 16.6% (103/620) of the total articles. Fourth in the ranking is the National Database of Health Insurance Claims and Specific Health Checkups of Japan (NDB) data, which was established by the MHLW in 2009, covering almost the whole population in Japan [1,2]. NDB was used in 10.5% (65/620) of the total articles.

#### Disease

According to the information on diseases in Table 1, we found that most studies have focused on infections (105/620, 16.9%), cardiovascular diseases (100/620, 16.1%), neoplasms (78/620, 12.6%), and nutritional and metabolic diseases (75/620, 12.1%). In addition, there were a number of studies on psychiatric disorders, indicated here as nervous system diseases (62/620, 10%) and mental disorders (38/620, 6.1%).

#### Outcome

The results of outcome show that treatment patterns (218/620, 35.2%), physiological or clinical outcomes (184/620, 29.7%), and mortality (137/620, 22.1%) were the most assessed outcomes. Comparatively, few (32/620, 5.2%) articles assessed guideline adherence. Only few studies measured quality indicators (5/620, 0.8%).

#### Statistical Method

Table 1 also suggests that most (414/620, 66.8%) studies were performed using multivariate modeling. In addition, we investigated the counts and percentages of modeling purposes (Figure 1) and specific models used in the 414 multivariate modeling studies in Table 2. The results show that most (375/414, 90.6%) of the multivariate modeling studies were performed for confounder adjustment. Some were conducted for clustered data modeling (69/414, 16.7%) and factor exploration (68/414, 16.4%). Two types of models were used for clustered data modeling: the generalized estimating equations (GEE) method and multilevel models. The GEE methods adjust for the clustering nature of the data and correctly estimate the SE of the estimated parameters. Multilevel models are often used with random effects to estimate the predictor effects for patients in specific clusters. Our results indicate a greater tendency to use multilevel regression (43/414, 10.4%) than GEE (26/414, 6.3%) in clustered data modeling studies. Only a few (8/414, 1.9%) studies analyzed cost-effectiveness. Regarding the specific models used in the multivariate modeling studies, logistic regression (249/414, 60.1%), Cox proportional hazards regression (87/414, 21%), and linear regression (57/414, 13.8%) were the most used.

#### Diseases and Outcomes

**Figure 3:**
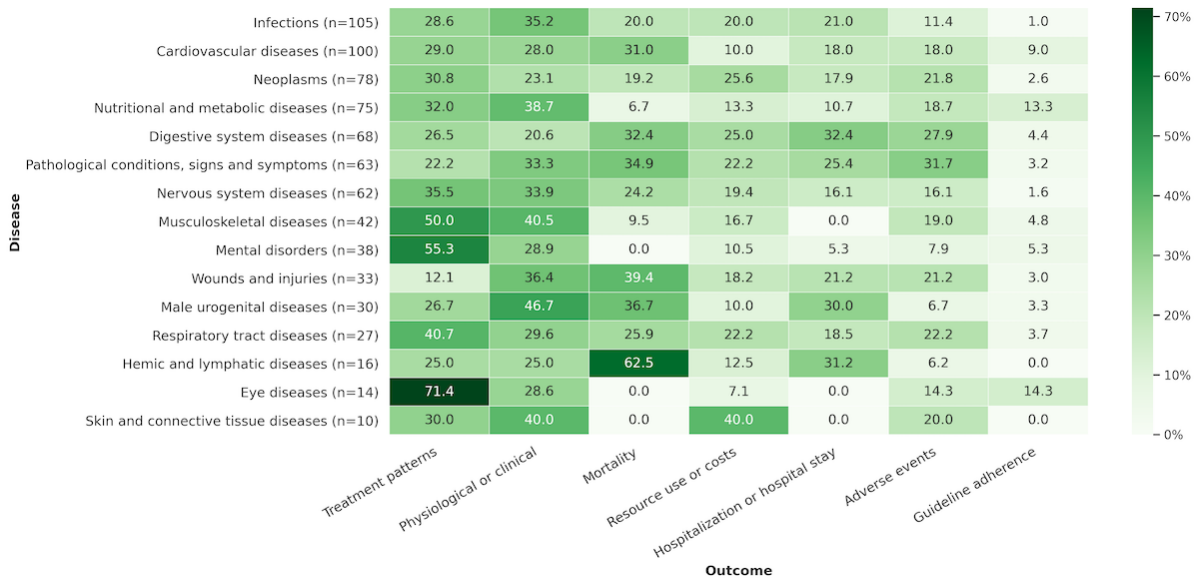
Percentages of outcomes in each disease.

We investigated the percentage of each outcome measured for different diseases. As shown in Figure 3, most (10/14, 71%) studies on eye diseases have focused on assessing their treatment patterns. Similarly, a number of studies on mental disorders (21/38, 55%), musculoskeletal diseases (21/42, 50%), and respiratory tract diseases (11/27, 40%) have also focused on assessing treatment patterns. Among the studies on hemic and lymphatic diseases, mortality accounted for the highest percentage (10/16, 63%), whereas few studies assessed adverse events. Furthermore, mortality has not been assessed in studies of mental disorders, eye diseases, and skin and connective tissue diseases. In addition, no study has assessed hospitalization or hospital stay in musculoskeletal, eye, and skin and connective tissue diseases.

#### Statistical Methods and Outcomes

**Figure 4:**
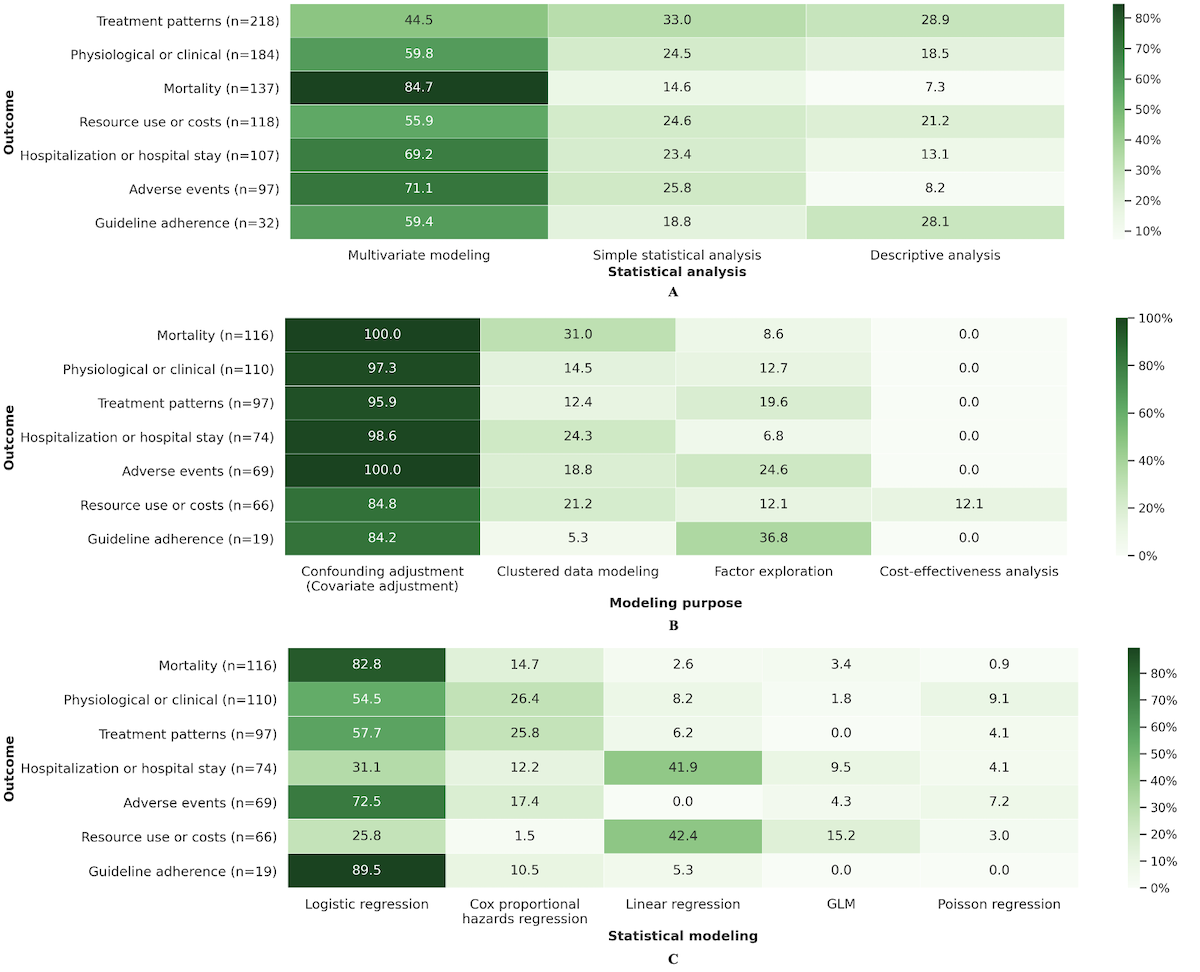
(A) Percentages of statistical analysis types for each outcome, (B) modeling purposes for each outcome, and (C) specific models for each outcome. GLM: generalized linear model.

We also calculated the percentages of statistical methods used to assess different outcomes. Figure 4A shows the percentages of the 3 types of statistical analyses used for each outcome; Figure 4B shows the percentages of multivariate modeling studies for different purposes for assessing these outcomes, and Figure 4C shows the percentage of each detailed multivariate model used for these outcomes. Multivariate modeling was used most frequently to assess mortality (116/137, 84.7%). Although the treatment patterns were the most assessed by the target studies (n=218), not many of them used multivariate modeling (97/218, 44.5%). Figure 4B indicates that almost all outcomes were measured with confounding adjustments. As shown in Figure 4C, logistic regression was the first choice for assessing mortality (96/116, 82.8%), physiological or clinical outcomes (60/110, 54.5%), treatment patterns (56/97, 58%), and guideline adherence (17/19, 90%). The results also suggest the use of Cox proportional hazards regression to assess these outcomes. In contrast, linear regression was the most commonly used model for assessing hospitalization or hospital stay (31/74, 42%) and resource use or costs (28/66, 42%).

## Discussion

### Principal Findings

A comprehensive narrative literature review was conducted to understand the secondary use of nationwide claims data, EMRs data, and EHRs data in clinical epidemiology in Japan. On the basis of the search strategy and eligibility criteria, a total of 620 eligible articles were identified from PubMed between January 1, 2006, and June 30, 2021 (the date of search).

We quantified 7 categories of key information from these 620 eligible articles. The main findings were that (1) most of the research has been done by academic institutions, whereas nonacademic institutions tend to collaborate with academic institutions; (2) the cohort study was the major design that longitudinally measured outcomes of proper patients; (3) most studies used claims data; (4) the JMDC, DPC database (MHLW), MDV, and NDB were mostly used, whereas only a few studies used EMRs or EHRs from a single hospital or multiple hospitals, which do not have a large patient population; (5) the top rank of diseases studied in the current research were infections, cardiovascular diseases, neoplasms, and nutritional and metabolic diseases; (6) treatment patterns, physiological or clinical outcomes, and mortality were the most assessed in these articles; and (7) multivariate models were commonly used, during which logistic regression and linear regression were shown to be the first choice for analyzing categorical variables and continuous variables, respectively.

The findings on the percentage of outcomes for different diseases hint at the tendency of existing studies to examine different diseases. For some common, chronic, and psychiatric diseases, current studies tended to assess their treatment patterns, whereas for some sudden onset severe diseases, patient mortality and hospitalization or hospital stay were assessed more often. Existing studies have focused more on assessing treatment modalities, physiological or clinical outcomes, and mortality when targeting diseases such as infections, cardiovascular diseases, and neoplasms. Furthermore, although strong trends were detected between eye diseases and treatment patterns, hemic and lymphatic diseases versus mortality, and mental disorders versus mortality (Figure 3), it was difficult to draw any conclusions that reflect clinical importance because of the small sample size. However, these results indicated different distributions of outcomes measured in different diseases, from which we can learn the focus and shortcomings of the existing studies. In addition, the total number of studies measuring guideline adherence was relatively small (n=32). During this period, 63% (20/32) of the studies were conducted on “cardiovascular diseases” and “nutritional and metabolic diseases.” These results also revealed a relative lack of studies measuring guideline adherence in infections. We expect that RWD research on guideline adherence would receive more attention in future.

The percentage of databases used for different diseases implied the selection of databases for observing different diseases. The JMDC databases and DPC database (MHLW) showed opposite use trends in diseases, especially nutritional and metabolic diseases, musculoskeletal diseases, mental disorders, hemic and lymphatic diseases, eye diseases, and skin diseases.

According to the investigation of statistical methods used to assess different outcomes, multivariate models were the most commonly used in assessing mortality. Regardless of the outcome, multivariate modeling was accompanied by adjustments for various confounders (Figure 4B). Mortality, hospitalization or hospital stay, and resource use or costs have been analyzed using multilevel models or marginal models (eg, GEE) more than others. This implies that hospital-related outcomes tended to be assessed by models that took clustering into account. Logistic regression was the first choice for measuring many of the outcomes, with the exception of hospitalization or hospital stay and resource use or costs, for which linear regression was commonly used. Cox proportional hazards regression was suggested as the second choice when assessing mortality, physiological or clinical outcomes, and treatment patterns. Although the PS technique has been proven effective in balancing confounders between groups, it has not been widely used in existing studies. There is a relative preference for this technique in studies assessing mortality.

### Comparison With Prior Work

In this subsection, we compare this review with 2 similar studies [27,28]. Hirose et al [27] conducted a narrative review of 68 studies on the secondary use of claims data in a specific database, NDB, from October 2016 to June 2019. They summarized 5 key pieces of information, including study design, research area, setting or sample, outcomes, and strengths and limitations. Subsequently, Fujinaga and Fukuoka [28] conducted a similar narrative review of 643 studies on the secondary use of claims data in 4 large-scale domestic databases: NDB, DPC database (MHLW), JMDC, and MDV, from January 2015 to October 2020, from which 3 categories of research type, design, and area were analyzed descriptively. Both studies used a classification of the journals in which the target articles were published to extract information about the research area [29]. These classifications mixed disciplinary categories, such as clinical medicine, pharmacology and pharmacy, pharmacology and toxicology, and immunology; disease categories, such as infectious diseases; and general categories, such as social sciences and public environmental health. In addition, only the primary outcomes were analyzed in these 2 studies. As a result, the distribution of articles in each category was summarized in these studies.

Because of the partial overlap in search periods, as well as the fact that PubMed was used for the search, there were some articles that were reviewed in both this study and these 2 prior studies. In contrast to these 2 studies, which used 1 or more specific claim databases without specifying a research area, our review investigated domestic epidemiological studies based on the secondary use of 3 types of RWD: claims, EMRs, and EHRs. A further difference is that we defined 7 categories for data collection to assess the status and trends of the existing studies. One of the novelties is that we classified the outcomes with reference to the paper by Abaho et al [24] paper and collected information on all the outcomes measured in the target articles. The advantage of this classification is that these outcomes are also applicable to clinical trial studies and can be automatically identified from biomedical articles [24]. Another innovative point is that we proposed a hierarchical approach to classify the statistical methods that appear in the target articles. For the results of the data collection, we summarized the distribution of the target articles in each category. Additional comparative analyses were performed for diseases versus outcomes (Figure 3), outcomes versus statistical methods (Figure 4), and diseases versus databases (Multimedia Appendix 5), which revealed trends in the assessment of outcomes across different diseases, trends of statistical methods used for different outcomes, and trends in database selection when analyzing different diseases. Moreover, our findings shed light on the focus and shortcomings of previous studies.

In addition, we identified several other review studies on the secondary use of RWD data [30-32]. The paper by Ferver et al [30] provided a narrative review of 1956 claims-based studies in 5 health care journals from 2000 to 2005 by summarizing the research types and areas. The paper by Hutchings et al [31] provided a systematic literature review of 18 studies to investigate the attitudes of relevant practitioners toward the secondary use and sharing of health administrative and clinical trial data. Schlegel et al [32] conducted a literature review of 941 studies on the secondary use of health care data in 2016 to select the best performing articles. We summarized these additional studies to understand other investigations on the secondary use of RWD data. Comparisons were not made because of the survey years or different research purposes.

### Limitations

The first limitation of this review is that we only searched the literature in PubMed, which may have led to significant publication bias. Second, we only investigated studies conducted in Japan. In the future, a comparison of studies from other countries, such as the United States, will be necessary to understand the Japan-specific trends of such studies. In addition, searches of multiple electronic databases should be considered to reduce potential publication bias.

### Future Directions

In this subsection, we discuss the future perspectives for the use of claims, EMRs, and EHRs in epidemiology in the Japanese context, in terms of the findings of this large narrative literature review.

#### Organization

Regarding collaborative aspects, with strong national promotion for RWD use and high level of interest from health care firms, collaborative research, involving multiple stakeholders and academic researchers, is seen to be necessary to leverage academic results and accelerate clinical applications.

#### RWD Type

Notably, only a few studies have used EHRs. EHRs have not been widespread in Japan because of the high cost of implementation and the difficulties in bridging different EHR service vendors. With the promotion of “cloud-based EHR” development by the Japanese Ministry of Internal Affairs and Communications, EHRs are expected to become widely used in the future.

#### Disease

With regard to the disease trend detected in this review, we made a rough comparison with worldwide trends. As we did not find a quantitative survey of RWD research on different diseases, the worldwide trend was roughly estimated by counting the number of related publications for different diseases. We focused on the top-ranked disease areas identified in this review, including infections, cardiovascular diseases, and neoplasms. The number of publications for these diseases was obtained by searching for electronic databases, such as PubMed or PubMed Central with search keywords: combinations of “claims,” “EHR,” “EMR,” to “infection,” “cardiovascular disease,” and “cancer.” We retrieved 18,847 publications on cancer, 7517 publications on infections, and 6624 publications on cardiovascular diseases from PubMed. The same trend was detected in PubMed Central. According to these counts, we estimated that the worldwide trend of the disease examined in existing studies was cancer. In contrast, our results revealed a Japan-specific trend in the studies on infections.

It is important to note that the above counts may be subject to bias because we have not designed any eligibility criteria for the precise search of related publications worldwide. In the future, it will be necessary to compare relevant studies with those of other countries to clarify the Japan-specific status and challenges.

#### Statistical Method

On the basis of the statistical skills used in the eligible articles, we summarized the appropriate statistical methods for use under different conditions. First, to design simple statistical analyses, our findings suggest using Fisher’s exact tests or chi-square test to compare categorical variables, and 2-tailed *t* test, ANOVA, and Mann-Whitney *U* test were used to compare continuous variables [33-36]. To evaluate variable change trends, the Cochran-Armitage test was used for categorical variables, whereas the Jonckheere-Terpstra test was used for continuous variables [37].

Suggestions for statistical methods to measure different outcomes are summarized in Table 3. For confounding adjustment, there are 2 methods: covariate adjustment and PS analysis. PS analysis is known to be an effective technique for balancing the patient backgrounds between the 2 groups across all putative risk factors or confounders [38-40]. However, referring to the study by Elze et al [41] that PS analysis is not necessarily superior to conventional covariate adjustment, we suggest selecting PS analysis with caution for confounder adjustment. Our findings also demonstrated that most existing studies used covariate adjustment (n=279) rather than PS analysis (n=96; Multimedia Appendix 4). In addition, hospital-based medical data are frequently clustered within medical centers or physicians. For instance, patients treated in a particular hospital may be more alike than those treated in another hospital because of differences in treatment policies. To model such clustered data, multilevel models with random effects have been suggested for use in estimating predictor effects for patients in specific clusters [42,43].

**Table 3:** Suggestions of statistical methods for measuring different outcomes.

Outcome	Method recommendation
Treatment patterns	Logistic regression, Cox proportional hazards regression
Physiological or clinical	Logistic regression, Cox proportional hazards regression
Mortality	Kaplan-Meier analysis, log-rank test, logistic regression, Cox proportional hazards regression
Hospitalization or hospital stay	Linear regression, GLM^a^
Adverse events	Logistic regression, Cox proportional hazards regression
Resource use or costs	Linear regression, GLM
Guideline adherence	Logistic regression
Quality indicators	Logistic regression

^a^GLM: generalized linear model.

In contrast, there were few studies on predictive machine learning models in this review (n=3; Multimedia Appendix 4). However, we roughly retrieved 2223 publications worldwide on PubMed by searching for the keywords of “claims,” “EHR,” “EMR,” and “machine learning.” Notably, we did not design any eligibility criteria for this study. The large difference in the number of articles indicates that epidemiological research based on claims, EMRs, and EHRs in Japan is backward in the use of artificial intelligence techniques.

### Conclusions

This literature review provides a good understanding of the current status and trends in the use of claims, EMRs, and EHRs in clinical epidemiology in Japan. The results demonstrated appropriate statistical methods regarding different outcomes, Japan-specific trend of disease areas, and lack of use of artificial intelligence techniques in existing studies. We hope that the results of this narrative review will provide useful information for researchers to design relevant studies. In the future, a more precise comparison of relevant domestic research with worldwide research will be conducted to clarify the Japan-specific status and challenges.

## Appendix

Multimedia Appendix 1 PubMed search string.

Multimedia Appendix 2 Explanation of the 7 categories of information.

Multimedia Appendix 3 Distribution of publication years of the 620 eligible articles.

Multimedia Appendix 4 Full results of counts and percentages of the 7 categories.

Multimedia Appendix 5 Percentages of databases used in each disease.

## Abbreviations

DPC: diagnosis procedure combination
EHR: electronic health record
EMR: electronic medical record
GEE: generalized estimating equations
GLM: generalized linear model
JMDC: Japan Medical Data Center Claims
MDV: medical data vision
MeSH: Medical Subject Headings
MHLW: Ministry of Health, Labour and Welfare
NDB: National Database of Health Insurance Claims and Specific Health Checkups of Japan
PRISMA: Preferred Reporting Items for Systematic Reviews and Meta-Analyses
PS: propensity score
RWD: real-world data

## Data Availability

All data generated or analyzed during this study are included in this published paper and its multimedia appendices.
