# Supplementary material for: The Current Status of Secondary Use of Claims, Electronic Medical Records, and Electronic Health Records in Epidemiology in Japan: Narrative Literature Review
Source: JMIR Med Inform. 2023 Feb 14;11:e39876. doi: 10.2196/39876 (PMC9975931; doi:10.2196/39876)
Supplement: Multimedia Appendix 1 [file medinform_v11i1e39876_app1.docx]

## Multimedia Appendix 1: PubMed search string

(("real world"[tiab] OR "database"[tiab]) AND ("claim*"[tiab] OR "receipt*"[tiab] OR "administrative"[tiab] OR "emr"[tiab] OR "ehr"[tiab] OR "electronic medical record*"[tiab] OR "electronic health record*"[tiab] OR "Electronic Health Records"[MAJR] OR "Administrative Claims, Healthcare"[MAJR] OR "Insurance Claim Review/statistics and numerical data"[MAJR])) AND ("japan*"[tiab] OR "Japan/epidemiology"[MeSH]) AND (("2006/01/01"[Date - Publication] : "2021/06/30"[Date - Publication]))
