# Supplementary material for: The Current Status of Secondary Use of Claims, Electronic Medical Records, and Electronic Health Records in Epidemiology in Japan: Narrative Literature Review
Source: JMIR Med Inform. 2023 Feb 14;11:e39876. doi: 10.2196/39876 (PMC9975931; doi:10.2196/39876)
Supplement: Multimedia Appendix 2 [file medinform_v11i1e39876_app2.docx]

## Multimedia Appendix 2: Explanation of the seven categories of information.

| **Category** | | **Explanation** |
| --- | --- | --- |
| **Organization** | |  |
|  | Academic | Studies conducted by research non-profit organizations such as universities, national institutes, etc. |
|  | Nonacademic | Studies conducted by non-academic enterprises including pharmaceutical companies, biotechnology companies, medical device companies or other healthcare-related companies. |
|  | Collaboration | Studies conducted by a collaboration of academic and non-academic institution. |
| **Study design** | |  |
|  | Cohort study | Studies in which patient cohorts with a common characteristic are followed longitudinally to assess a certain health outcome (disease, condition, event, or a change in health status or behavior). |
|  | Case-control study | Studies in which patients are compared between individuals with a disease/condition (cases) and comparable individuals who do not have the disease/condition (controls). |
|  | Case-crossover study | Studies in which patients are from the same case group with different time periods to identify intermittent exposure information. |
|  | Cross-sectional study | Studies that observe a patient population at a single time point. |
| **RWD type** | |  |
|  | Claim | Known as administrative claims, composes of doctors' appointments, bills, prescriptions, insurance information, and other patient-provider communications |
|  | EMR | Data from electronic medical records |
|  | EHR | Data from electronic health records |
| **Disease** | | Consists of 26 diseases representing MeSH code C01-C26. |
| **Database** | | RWD databases used in the target articles. |
| **Outcome** | |  |
|  | Treatment patterns | Includes drug prescription, drug switch, discontinuation, retreatment, and ongoing treatment. |
|  | Physiological or clinical | Includes measures of physiological function, signs and symptoms, scientific measures relating to physiology. |
|  | Adverse events | Includes outcomes broadly labeled as some form of unintended consequence of the intervention. |
|  | Mortality | Includes overall survival/mortality as well as composite survival outcomes that include death. |
|  | Resource use or costs | Includes economic outcomes and resource utilizations. |
|  | Hospitalization or hospital stay | Includes outcomes relating to inpatient or hospital care such as duration of hospital stays, admission to ICU. |
|  | Guideline adherence | Describes the extent to which a patient correctly follows medical advice. |
|  | Quality indicators | Describes measures of health care quality. |
| **Statistical method** | |  |
|  | Multivariate modeling | Involvement of multivariate statistical modeling analyses for the purpose of confounding adjustment, clustering data modeling, factor exploration, or cost-effectiveness analysis. |
|  | Simple statistical analysis | Involvement of significance tests (e.g. T-test, Chi-square test, Kaplan–Meier analysis, etc.) |
|  | Descriptive analysis | Involvement of descriptive statistics only (e.g. mean, standard deviation (SD), count, frequency, percentage, etc.). |
