# Supplementary figures and images for: The Current Status of Secondary Use of Claims, Electronic Medical Records, and Electronic Health Records in Epidemiology in Japan: Narrative Literature Review
Source: JMIR Med Inform. 2023 Feb 14;11:e39876. doi: 10.2196/39876 (PMC9975931; doi:10.2196/39876)

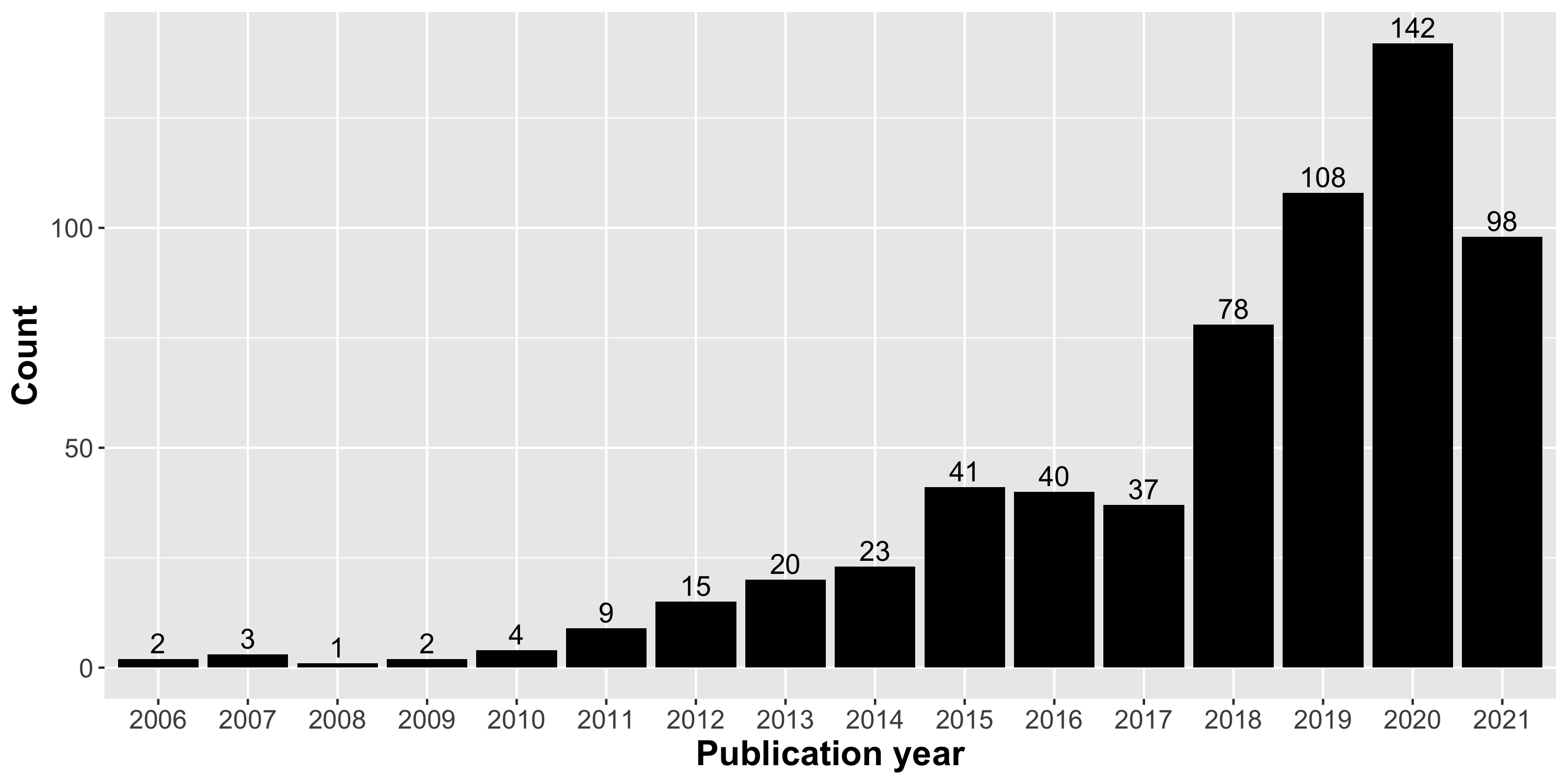

Supplement: Multimedia Appendix 3 [file medinform_v11i1e39876_app3.png]

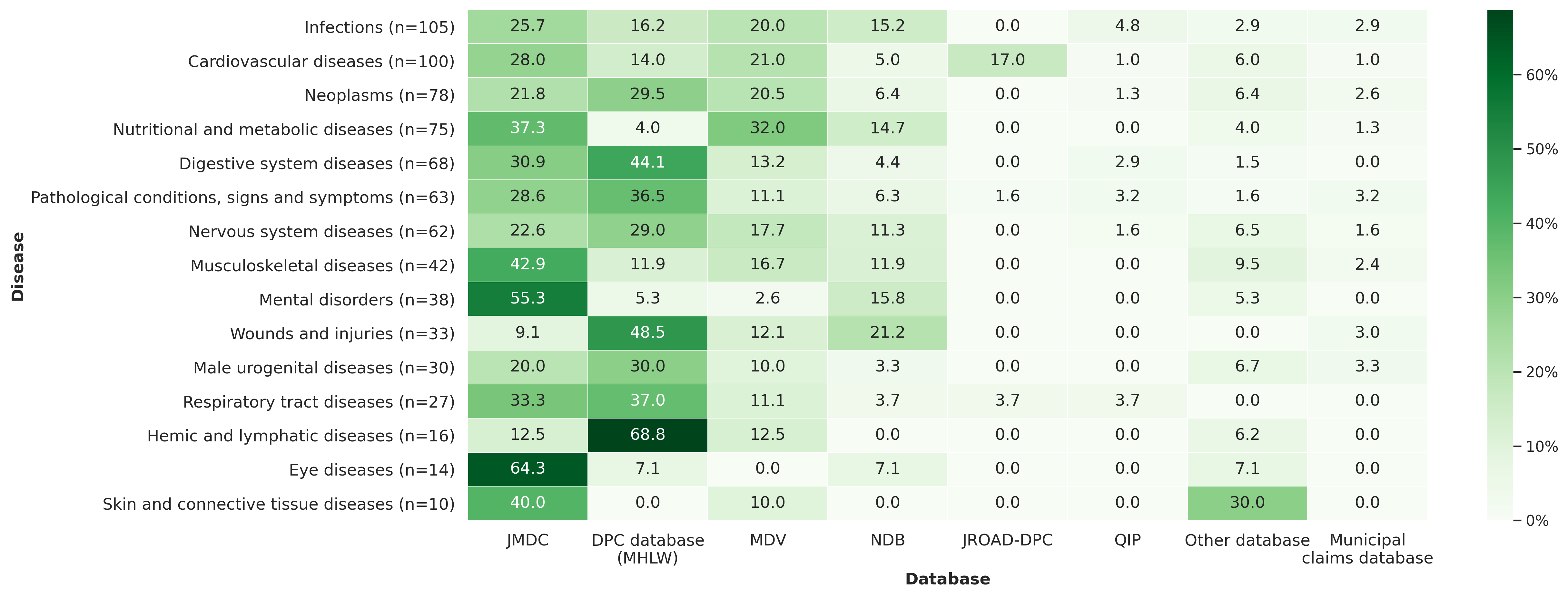

Supplement: Multimedia Appendix 5 [file medinform_v11i1e39876_app5.png]
